# Medical Student Burnout by Race, Ethnicity, and Multiple Disability Status

**DOI:** 10.1001/jamanetworkopen.2023.51046

**Published:** 2024-01-10

**Authors:** Mytien Nguyen, Lisa M. Meeks, Karina Pereira-Lima, Justin L. Bullock, Amy N. Addams, Christopher J. Moreland, Dowin B. Boatright

**Affiliations:** 1 Department of Immunobiology, Yale School of Medicine, New Haven, Connecticut; 2 Department of Learning Health Sciences, University of Michigan Medical School, Ann Arbor; 3 Department of Family Medicine, University of Michigan Medical School, Ann Arbor; 4 Department of Neurology, University of Michigan Medical School, Ann Arbor; 5 Department of Medicine, Division of Nephrology, University of Washington School of Medicine, Seattle; 6 Association of American Medical Colleges, Washington, DC; 7 Department of Internal Medicine, Dell Medical School at the University of Texas at Austin, Austin; 8 Department of Emergency Medicine, New York University Grossman School of Medicine, New York

## Abstract

This cohort study examines the prevalence of burnout among students underrepresented in medicine by race and ethnicity with multiple disability types.

## Introduction

Burnout is associated with depression and attrition in the physician workforce. Recent studies have found that Asian, Black, and Hispanic students and students with disabilities experience increased risks of burnout.^1,2^ However, little is known about the risk of burnout among racial and ethnic underrepresented students with a disability, or among students who have cooccurring disabilities. Here, we examined the prevalence of burnout among students underrepresented in medicine by race and ethnicity (URiM; American Indian or Alaska Native, Black, Hawaiian Native, Hispanic, Pacific Islanders) with multiple disability types.

## Methods

This cohort study was deemed exempt by the University of Colorado Medical School institutional review board because data were deidentified. Informed consent was obtained by the Association of American Medical Colleges (AAMC). We analyzed 2019 and 2020 deidentified student data from the AAMC Year 2 Questionnaire (Y2Q). Data included self-reported race, ethnicity, age, sex, and disability status. Self-reported disabilities were categorized into disability types (eMethods in Supplement 1), and categorized by number of reported disability types. We followed the STROBE reporting guideline.

High risk of burnout was classified as students who were in the top quartile among the study cohort for both the exhaustion and disengagement subscales of the Oldenburg Burnout Inventory for Medical Students (eMethods in Supplement 1).^1^ We used descriptive statistics to assess the prevalence of disability by race, ethnicity, sex, and age. Modified Poisson regression was used to estimate the relative risk of burnout by race, ethnicity, multiple disability status, and their intersections, adjusting for age, sex, and school-level prevalence of students with disabilities. Analyses were performed using Stata version 18.0 (StataCorp) from July to August 2023. Two-sided *P* < .05 was considered statistically significant.

## Results

Among 27 009 students who completed the Y2Q, 23 889 (88.48%) had complete disability and sex data and were included in the analyses (13 448 [56.29%] female, 5064 [21.29%] Asian, 3048 [12.75%] URiM, 1937 [8.11%] reported 1 disability type, and 466 [1.95%] reported multiple disability types) (Table). Compared with White students, a lower proportion of Asian students reported 1 disability type (4.25% vs 8.94%; *P* < .001) and multiple disability types (0.93% vs 2.21%; *P* < .001). URiM and White students reported similar prevalence of 1 disability type (9.12% vs 8.94%; *P* = .72) and multiple disability types (2.49% vs 2.21%; *P* = .33) (Table).

**Table.** Prevalence of Multiple Disability Status by Race, Ethnicity, Sex, and Age

Variables	Students, No. (%)			*P* value^a^
	0 Disabilities	1 Disability	≥2 Disabilities	
Total	21 486 (89.94)	1937 (8.11)	466 (1.95)	NA
Race and ethnicity				
Asian	4801 (94.83)	215 (4.25)	47 (0.93)	<.001
URiM	2693 (88.38)	278 (9.12)	76 (2.49)	
White	11 623 (88.85)	1169 (8.94)	289 (2.21)	
Other^b^	2369 (87.81)	275 (10.19)	54 (2.00)	
Sex				
Male	9451 (90.52)	832 (7.97)	158 (1.51)	<.001
Female	12 035 (89.49)	1105 (8.22)	308 (2.29)	
Age, y				
≤26	18 335 (91.16)	1462 (7.27)	317 (1.58)	<.001
>26	3151 (83.47)	475 (12.58)	149 (3.95)	

Abbreviations: NA, not applicable; URiM, underrepresented in medicine by race and ethnicity (Black, Hispanic, Hawaiian Native, Alaskan Native, or Pacific Islander).

^a^ *P* values were calculated using Pearson χ^2^ test.

^b^ Other race and ethnicity included multiracial students and students with unknown race and ethnicity.

A total of 3265 students (13.66%) were at high risk for burnout. Burnout risk increased with increasing number of disability types (2711 students without disabilities [12.61%], 408 students with 1 disability type [21.05%], and 146 students with multiple disability types [31.33%]). After adjusting for race, ethnicity, sex, age, and school-level prevalence of disability, students with 1 and multiple disabilities were at 70% and 254% greater risk of burnout than their peers, respectively (Figure). Intersectional analysis between race, ethnicity, and disability status found that Asian and URiM students with multiple disabilities had the highest burnout risk. Compared with White students without disabilities, Asian (adjusted risk ratio [aRR], 3.23 [95% CI, 2.21-4.72]) and URiM (aRR, 3.10 [95% CI, 2.26-4.25]) students with multiple disability types were at more than 3-fold greater risk of burnout (Figure); and compared with White students with 1 disability type, Asian (aRR, 1.31 [95% CI, 1.00-1.72]) and URiM (aRR, 1.30 [95% CI, 1.01-1.68]) students with 1 disability were at approximately 30% greater risk of burnout.

**Figure.**
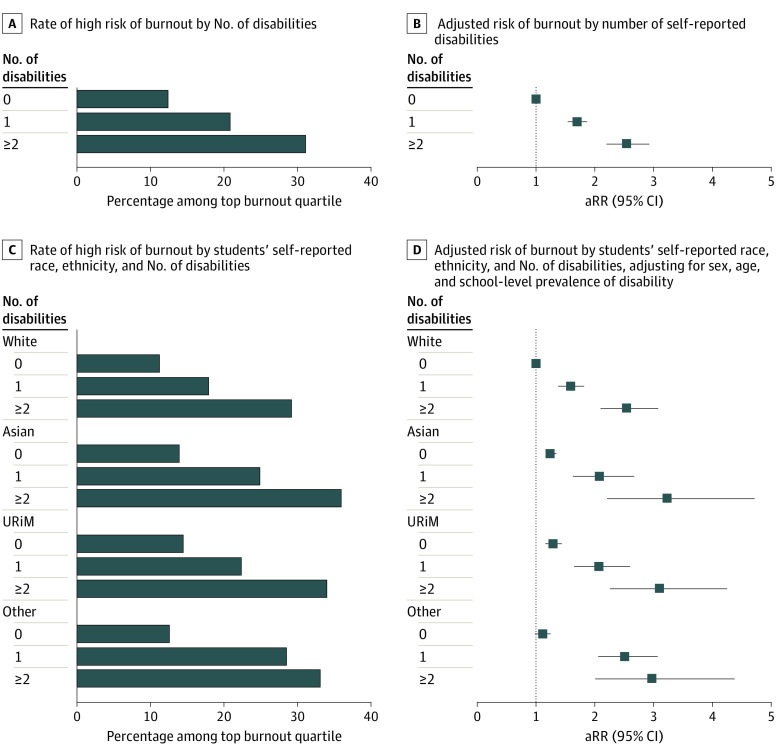
Rate and Adjusted Risk of Burnout by Student Demographics Other indicates multiracial students and students with unknown race and ethnicity; URiM , underrepresented in medicine by race and ethnicity (Black, Hispanic, American Indian or Alaska Native, Hawaiian Native, Pacific Islanders); other, multiracial students and students with unknown race and ethnicity.

## Discussion

In this cohort study, we found that Asian and URiM students with multiple disability types experienced the highest risk of burnout and were at more than 3-fold greater risk of burnout compared with their White peers without disability. For Asian and URiM students, who are already facing substantial mistreatment and discrimination,^1,3^ the allostatic load of having multiple disabilities was particularly detrimental. This highlights the importance of addressing the needs of students with disabilities through an intersectionality lens in medical training,^4^ particularly those from underrepresented backgrounds with multiple disability types.

Access to accommodations have shown to improve burnout metrics among students with disabilities.^2^ Fear of stigma and lack of clear institutional processes are substantial barriers to disability disclosure and requesting accommodations.^5^ Limitations of this study include our inability to cluster results by medical school and to examine prevalence of multiple disabilities and burnout across other demographic groups, including sexual and/or gender minority^2^ and socioeconomic status.^6^ This study highlights the necessity to address burnout among Asian and URiM students with multiple disabilities through applying critical intersectional, antiracist, and antiableist lenses to promote equity in medical training.

## Notes

Supplement 1. **eMethods.** Supplemental Methods

Supplement 2.
Data Sharing Statement
