# Supplementary material for: Medical Student Burnout by Race, Ethnicity, and Multiple Disability Status
Source: JAMA Netw Open. 2024 Jan 10;7(1):e2351046. doi: 10.1001/jamanetworkopen.2023.51046 (PMC10782232; doi:10.1001/jamanetworkopen.2023.51046)
Supplement: Supplement 1. — eMethods. Supplemental Methods [file jamanetwopen-e2351046-s001.pdf]

Supplemental Online Content

Nguyen M, Meeks LM, Pereira-Lima K, et al. Medical student burnout by race, ethnicity, and multiple disability status. *JAMA Netw Open.* 2024;7(1):e2351046. doi:10.1001/jamanetworkopen.2023.51046

eMethods. Supplemental Methods

This supplemental material has been provided by the authors to give readers additional information about their work.

© 2024 Nguyen M et al. *JAMA Network Open.*

eMethods. Supplemental Methods

Race and ethnicity were assessed because burnout has been shown to be higher among racial and ethnic marginalized students in prior studies.[1] Students self-reported identifying as Black, Hispanic, Hawaiian Native, Alaskan Native, Pacific Islander or Other. Students who reported selected multiple racial categories or who did not respond were re-categorized as "Other." Students identifying as Black, Hispanic, Hawaiian Native, Alaskan Native, or Pacific Islander were categorized as underrepresented in medicine by race and ethnicity (URiM). The Association of American Medical Year-2 Questionnaires (Y2Q) asks students to describe your disability, "*Which of the following best describes your disability? If you have more than one type, select all that apply. [Optional: please add a brief description where provided].*" The choices included: Attention deficit/hyperactivity disorder (ADHD), chronic health disability, dead or hard of hearing, learning disability, motility disability, psychological disability, visual disability, and other. Students in others were recategorized to one of the following categories based on their free response: ADHD, chronic illness, hearing, vision, learning, mobility/physical, neurological, psychological, speech, and Autism Spectrum Disorder (ASD)/Asperger's. ASD and Asperger's were prevalent in the cohort and was assigned their own disability type.

The Oldenburg Burnout Inventory for Medical Students is a 16-questions survey with two subscales for exhaustion and disengagement dimensions of burnout. Exhaustion refers to the cognitive and physical fatigue and disengagement describes the distancing from and negative attitudes toward the objects or contents of medical education. Each dimension is assessed

© 2024 Nguyen M et al. *JAMA Network Open.*

through eight questions on a scale from 0 to 3, and responses were transformed so that higher scores corresponded with increased burnout. Thus, each dimension has a score ranging from 0 to 24. We determined high risk of burnout as students who were in the top quartile among the study cohort for both the exhaustion (scores 16 and above) and disengagement (scores 12 and above) subscales of the Oldenburg Burnout Inventory for Medical Students.

References

1. Teshome BG, Desai MM, Gross CP, et al. Marginalized identities, mistreatment, discrimination, and burnout among US medical students: cross sectional survey and retrospective cohort study. *BMJ*. 2022;376:e065984. doi:10.1136/bmj-2021-065984

© 2024 Nguyen M et al. *JAMA Network Open.*
