# Supplementary material for: Medical Student Burnout by Race, Ethnicity, and Multiple Disability Status
Source: JAMA Netw Open. 2024 Jan 10;7(1):e2351046. doi: 10.1001/jamanetworkopen.2023.51046 (PMC10782232; doi:10.1001/jamanetworkopen.2023.51046)
Supplement: Supplement 2. — Data Sharing Statement [file jamanetwopen-e2351046-s002.pdf]

Data Sharing Statement

Nguyen. Medical Student Burnout by Race, Ethnicity, and Multiple Disability Status. *JAMA Netw Open*. Published January 10, 2024. doi:10.1001/jamanetworkopen.2023.51046

Data
Data available: No
